# AI-based interviews and applicant perceptions: insights from China and Pakistan

**DOI:** 10.3389/fpsyg.2026.1886963

**Published:** 2026-08-25

**Authors:** GuangJun Zhao, Qiaoli Liang, Fahad Alam, Md Billal Hossain

**Affiliations:** 1 Business School, Shandong Management University, Jinan, Shandong, China; 2 School of Business, Beijing Geely University of China, Beijing, China; 3 School of Economics and Management, Tongji University, Shanghai, China; 4 Sustainability Competence Centre, Széchenyi Istvàn University, Győr, Hungary

**Keywords:** AI-based interview, interactive communication, job pursuit intention, trust in AI system, cross-culture

## Abstract

**Introduction:**

Artificial intelligence (AI) technology is increasingly integrated into recruitment processes, yet cross-cultural differences in job applicant responses to AI-based interviews remain understudied. Drawing on Signaling Theory and Fairness Heuristic Theory, this study examines how applicants in China and Pakistan perceive AI-based versus human-based interviews, focusing on three critical outcomes: perceived transparency, interactive communication, and job pursuit intention. It further examines whether trust in AI moderates these relationships and whether this moderating role is comparable across the two cultural contexts.

**Methods:**

Data were collected from 225 respondents in China and 213 respondents in Pakistan using a convenience sampling technique. Partial Least Squares Structural Equation Modeling was used to test the proposed relationships, followed by measurement invariance assessment and multi-group analysis.

**Results:**

The results show that AI-based interviews were negatively associated with perceived transparency, interactive communication, and job pursuit intention, with stronger negative associations observed among respondents in the Pakistani sample than in the Chinese sample. Moreover, trust in AI systems emerged as a significant positive moderator in both cultural contexts, suggesting that higher trust in AI weakened negative applicant responses to AI-based interviews.

**Discussion:**

Overall, the findings enhance understanding of how interview type and related perceptions shape applicant attitudes in cross-cultural recruitment settings. The study provides practical implications for HR managers and organizations, and discusses key findings, managerial recommendations, study limitations, and directions for future research.

## 1 Introduction

Organizations are increasingly transitioning from traditional recruitment methods to artificial intelligence (AI) technologies, particularly in cross-cultural contexts (Kulal et al., 2025; Sandeep et al., 2025). AI is implemented across hiring stages, including resume screening, professional record analysis, and conducting interviews (Balcioğlu and Artar, 2024). This enables organizations to evaluate diverse applicant data, including personality traits (Van Esch et al., 2019), nonverbal behavior (Park and Jung, 2025), and employment history (Malik et al., 2023a). Beyond data collection, AI-based recruitment enhances efficiency by reducing hiring time, increasing productivity, and lowering costs (Park and Jung, 2025). However, despite operational advantages, critical questions remain regarding how applicants, the primary stakeholders, perceive and respond to AI-driven selection methods across diverse cultural contexts.

Despite global adoption of AI-based hiring, applicant responses to AI-based interviews remain underexplored. In particular, cultural differences in applicants’ behaviors and attitudes toward AI-driven interviews have received limited empirical attention (Hoang et al., 2012; Park and Jung, 2025). As individual behavior is closely tied to cultural norms and values, these factors influence applicants’ perceptions, cognitive responses, and interpretations of technological tools in the recruitment process (Mantello et al., 2023; Tsai et al., 2011). Existing studies on AI-based interviews have been conducted in Western contexts, particularly in the United States and Europe (Folger et al., 2022; Rauf et al., 2021), suggesting applicants perceive AI systems as impersonal and unfair, which in turn undermines trust in the recruitment process and challenges organizational hiring outcomes. However, the present study does not directly test a Western versus non-Western comparison. Instead, the predominance of Western evidence is used as a starting point to examine whether applicant responses to AI-based interviews vary within non-Western Asian contexts (Park and Jung, 2025). Specifically, this study compares China and Pakistan, two broadly collectivistic societies that differ meaningfully in technological development (Alam et al., 2024), AI familiarity, institutional context, and recruitment expectations. This design allows the study to move beyond the simple Western and non-Western distinction and examine how variation within Asian contexts may shape applicant responses to AI-based interviews.

Three critical research gaps limit both theoretical understanding and practical application. First, existing literature exhibits a Western-centric bias (Folger et al., 2022; Rauf et al., 2021), with applicant responses in non-Western cultures, particularly Asian developing economies, largely unexplored. This creates uncertainty about whether negative AI perceptions represent broadly shared human responses or culturally contingent reactions. Second, we lack theoretical clarity regarding which technology acceptance mechanisms operate across the Chinese and Pakistani contexts and which are more context-specific. While trust drives technology acceptance (Glikson and Woolley, 2020), empirical evidence comparing its operation across substantially different cultural contexts remains limited. Third, organizations implementing AI recruitment globally lack evidence-based guidance for standardization versus localization decisions, creating implementation risks, including adverse impacts on employer branding and talent acquisition effectiveness.

Addressing these gaps requires systematic cross-cultural comparison between contexts differing meaningfully in cultural values and technological development. Existing cross-cultural recruitment studies predominantly compare Western individualistic cultures with East Asian collectivistic cultures (Park and Jung, 2025), leaving intra-Asian comparisons largely unexplored. Previous studies suggest that individuals’ behaviors, attitudes, and perceptions are shaped by cultural norms and values (Abdalla et al., 2024; Fell and König, 2016; König et al., 2021; Ma and Kang, 2020; Park and Jung, 2025). In AI-based interviews, job seekers often exhibit negative perceptions during initial recruitment stages (Suen et al., 2019). Individuals tend to trust human-based interviews more regarding perceived transparency, as diverse cultural backgrounds interpret new technologies through cultural lenses (Chien et al., 2025; Park and Jung, 2025). In cultures where personal control is less emphasized and direct social interaction is limited, AI-based interviews may be perceived as less fair (Park et al., 2021). Han et al. (2025) argued that individual social interactions vary across cultures, suggesting that fairness expectations and interpersonal cues in AI-based interviews cannot be universally applied. Applicants develop initial job and organizational perceptions through first interactions (e.g., interviews) (Nørskov et al., 2022), crucially shaping overall impressions. A negative experience with an AI-based interview may therefore diminish applicants’ job pursuit intention (Park and Jung, 2025). Although existing studies have explored cultural influences on individual attitudes and behaviors, revealing both similarities (Hanel et al., 2018) and differences across cultures (Persson et al., 2021), specific mechanisms through which culture shapes AI acceptance in recruitment contexts remain theoretically underdeveloped and empirically underexamined.

Furthermore, trust has been widely recognized as a fundamental factor influencing individual perceptions and behaviors across various domains, including technology adoption (e.g., AI) (Glikson and Woolley, 2020; Lee and Rich, 2021; Montag et al., 2024). In organizational settings, applicants respond more positively to recruitment processes when trusting technological systems (Suen and Hung, 2023). Trust is central to numerous ethical AI guidelines established by international organizations (Cannarsa, 2021; Smuha, 2019). Previous research highlighted AI benefits in human resource management, including enhanced performance evaluations (Varma et al., 2024), training and development (Maity, 2019), and job-related efficiency (Joshi and Masih, 2023). However, a notable gap remains in understanding the role of trust in AI systems in shaping applicant behavior across different cultural contexts. Specifically, while trust is theoretically positioned as a core mechanism in technology acceptance models (Venkatesh et al., 2003), empirical evidence examining whether trust operates similarly across substantially different cultural recruitment contexts is scarce. This represents both a theoretical gap, regarding cultural boundedness versus consistency of trust mechanisms, and a practical gap regarding whether trust-building strategies require cultural adaptation.

The primary objective is to investigate AI-based interview impact on job applicant behavior within cross-cultural contexts through a dual theoretical lens, examining both culturally-specific reactions and more broadly shared psychological mechanisms. Specifically, this study compares potential job applicants’ attitudes and responses from China and Pakistan toward AI-based versus traditional human-conducted interviews. China and Pakistan represent theoretically appropriate comparison cases: both are Asian collectivistic societies, yet they differ substantially in technological infrastructure (Alam et al., 2024; Hofstede, 2001), AI familiarity, and cultural orientations toward technology adoption, making them well suited for examining the interplay between cultural similarity and technological context in shaping AI acceptance. First, the study examines how AI-based interviews influence applicants’ perceptions of transparency, interactive communication, and job pursuit intention across the two contexts. Second, it explores the moderating role of trust in AI systems in the relationship between AI-based interviews and applicant perceptions, examining whether this moderating role is consistent across the two cultural contexts studied. Third, by comparing two cultures at different development levels, the study provides evidence-based insights into whether AI recruitment practices require cultural localization or can be standardized across broadly similar cultural regions. Third, by comparing different cultures and development levels, the study provides evidence-based insights into whether AI recruitment practices require cultural localization or can be standardized across similar cultural regions.

The research employs Signaling Theory (Spence, 1973b) and Fairness Heuristic Theory (Lind, 2001) as theoretical foundations for analyzing how different interview formats affect applicant behavior across cultures. Signaling theory explains how recruitment method choices serve as organizational signals that applicants interpret through cultural lenses, while fairness heuristic theory provides the cognitive framework through which applicants form justice perceptions based on early procedural cues, both processes that should be culturally contingent (Bangerter et al., 2012). However, trust mechanisms may operate more universally as fundamental psychological processes underlying technology acceptance. The findings will contribute to a deeper understanding of cross-cultural perceptions of AI-based interviews and offer practical insights for multinational organizations designing and implementing AI-driven recruitment practices across diverse cultural environments.

## 2 Conceptual framework and hypothesis development

AI is defined as a system’s capability to interpret data, analyze it, make predictions, and achieve results through adaptive learning mechanisms (Pan and Froese, 2023). In recruitment contexts, AI-based interviews involve adopting AI tools to support screening, selection, and identification of suitable applicants (Lee & Kim, 2021b). Scholars emphasize evaluating technology’s specific design features rather than treating it as monolithic (Park et al., 2024; Sumathi et al., 2024). This study focuses on AI-based interviews in which AI-enabled tools are used to conduct, structure, record, or evaluate candidate responses during the interview process. In contrast, traditional human-led interviews refer to interviews in which human recruiters or managers directly interact with applicants, ask questions, assess responses, and make evaluative judgments. Importantly, the use of AI in recruitment does not necessarily imply the complete absence of human managers, as organizations may still retain human oversight or final decision-making authority. Rather, the key distinction in this study lies in the extent to which the interview experience is mediated by algorithmic assessment rather than direct interpersonal interaction. This conceptual distinction is important because AI-based interviews may shift applicants’ experience from primarily social interaction toward technology-mediated evaluation, potentially altering psychological expectations between applicants and organizations (Rousseau, 1995) and shaping justice perceptions emerging from procedural cues (Lind, 2001). AI technology use during interviews represents a novel approach, garnering attention and is perceived by applicants as a signal about organizational hiring processes (Yu et al., 2025). Throughout recruitment, applicants interpret various cues and assign symbolic meanings (Park et al., 2021). According to signaling theory, cues from company websites, recruiters, or leadership serve as signals shaping applicants’ attitudes and behaviors toward organizations (Bangerter et al., 2012). Selection methods themselves convey symbolic values significantly influencing organizational perceived attractiveness (Park and Jung, 2025).

Signaling theory (Bangerter et al., 2012; Spence, 1973a) provides a foundational framework for understanding how recruitment practices communicate organizational characteristics to potential applicants. Organizations emit intentional and unintentional signals through selection procedures, which applicants interpret as indicators of organizational culture, values, and employee treatment (Celani and Singh, 2011). Choosing AI-based versus human-based interviews represents a particularly powerful signal, communicating information about organizational technological sophistication, modernization, and potentially its valuation of human interaction versus efficiency (Yu et al., 2025). However, signal interpretation is not universal but rather filtered through applicants’ cultural frameworks, prior experiences, and contextual understanding (Bangerter et al., 2012), creating potential for misinterpretation when organizations implement standardized AI recruitment across diverse cultural contexts without considering how different cultural groups decode technological signals.

Beyond signaling organizational characteristics, the fairness heuristic theory (Lind, 2001) explains cognitive processes through which applicants form justice judgments about recruitment procedures. Individuals form initial fairness impressions based on early procedural information, which then serve as heuristics for subsequent evaluations and behavioral intentions (Van den Bos et al., 2012). In recruitment contexts, interviews, as the first direct interactions between applicant and organization, provide critical procedural information shaping fairness perceptions (Gilliland, 1993). Crucially, fairness heuristic theory suggests initial justice impressions form rapidly and influence subsequent attitudes even when additional information becomes available (Lind, 2001). Therefore, if AI-based interviews create initial procedural unfairness impressions, through reduced transparency, limited interactive communication, or perceived algorithmic bias, these negative heuristics may persist and influence job pursuit intentions regardless of subsequent organizational efforts demonstrating fairness.

AI-based interview design and perception are influenced by contextual factors, including economic development and cultural norms (Park and Jung, 2025; Woo et al., 2024). In economically strong and technologically advanced countries like China, applicants exhibit more positive attitudes toward AI-based recruitment due to frequent exposure, familiarity, and confidence in using advanced technologies (Yan et al., 2025). China is a highly industrialized nation with strong innovation capacity, reflected in high GDP per capita and favorable global competitiveness rankings (Li, 2018). China’s extensive AI infrastructure investment, widespread AI adoption in consumer applications (facial recognition, mobile payments, recommendation systems), and government initiatives promoting AI development created a cultural environment characterized by high AI familiarity and generally positive attitudes toward algorithmic decision-making (Qin et al., 2020). This technological ecosystem may predispose Chinese applicants to interpret AI-based recruitment signals more favorably, viewing them as organizational modernity and efficiency indicators rather than threatening or impersonal (Li et al., 2025). In contrast, developing countries like Pakistan face substantial challenges in political instability, terrorism, and intercultural conflict, hindering economic development and technological advancement (van der Eng, 2025). Pakistan’s technological infrastructure remains less developed, with lower AI adoption rates in consumer and organizational contexts (Khurshid et al., 2024). This limited AI systems’ exposure may result in lower AI literacy and greater uncertainty regarding algorithmic decision-making, potentially amplifying fairness, transparency, and human interaction loss concerns in recruitment (Bibi, 2019). Additionally, Pakistani professional culture emphasizes interpersonal relationships and face-to-face communication in business contexts (Humaira Gul Saeed, 2016), which may conflict with the AI-based interviews’ impersonal nature and contribute to more negative signal interpretation.

Yousaf and Wu (2024) highlighted significant differences in cultural norms and values between China and Pakistan, particularly in collectivism, uncertainty avoidance, religion, and social structures. Pakistan exhibits relatively low employment rates and pronounced gender disparities (Rizvi et al., 2023), whereas China maintains comparatively higher employment rates (Xiang B. et al., 2023). These cultural differences influence how applicants perceive themselves and others (Park and Jung, 2025), shaping attitudes toward autonomous AI assessments and fairness perceptions within recruitment processes. Zheng et al. (2024) reported that individuals in developing economies with high technological development are more likely to view AI-based recruitment as credible and desirable. Applicants from economically advanced societies demonstrate greater adherence to social norms (Huang et al., 2023), correlating with more positive attitudes toward adopting new technologies (Zheng et al., 2024). Cultural values play a crucial role in shaping hiring practices and justice perceptions (Park and Jung, 2025). Han et al. (2025) emphasized that cultural values significantly impact fairness interpretations. Shao et al. (2013) found that cultural dimensions (individualism versus collectivism, uncertainty avoidance, power distance, achievement orientation) moderate relationships between organizational justice and job-related outcomes. Specifically, uncertainty avoidance is the extent to which societies feel threatened by ambiguous situations and create structures minimizing uncertainty (Merkin, 2006), may be particularly relevant in AI recruitment contexts. Pakistan scores higher on uncertainty avoidance compared to China (Yousaf et al., 2022), suggesting that Pakistani applicants may experience greater discomfort with the unpredictability and lack inherent in AI-based interviews. This cultural characteristic, combined with lower technological familiarity, may amplify negative reactions to algorithmic selection processes. Therefore, we propose that cultural differences between China and Pakistan will influence candidates’ perceptions of transparency, interactive communication, and job pursuit intentions in AI-based interview contexts. We examine trust in AI systems’ moderating role, affecting how attractive perceive the hiring organization.

### 2.1 Across-cultural individual perception of AI-based interviews and perceived transparency

Psychological attitude can be defined as a stable tendency developed through previous experiences that shapes an individual’s evaluation of specific objects or phenomena (Abdalla et al., 2024). In the context of AI, individuals’ attitudes are closely tied to their willingness to engage with and integrate AI technologies into daily life (Han et al., 2025). Understanding these attitudes is therefore essential for guiding the effective and ethically responsible use of AI systems across cultures. Mantello et al. (2023) reported that different cultural backgrounds play a different role in shaping individuals’ attitudes toward AI. For instance, Globig et al. (2024) found that individuals in the UK and USA exhibit varying perceptions of AI, viewing it as either a threat or a benefit, shaped by contextual factors such as culture, age, and gender. In a large-scale international survey involving over 10,000 respondents across eight countries, such as Australia, Canada, the USA, South Korea, France, Brazil, India, and Nigeria, Kelley et al. (2021) explored individual attitudes towards AI concerning four key dimensions: “exciting,” “useful,” “worrying,” and “futuristic.” The results revealed that participants from developed countries (e.g., USA, Canada, and Australia) predominantly associated AI with concern and futuristic implications. In contrast, respondents from developing countries (e.g., India, Brazil, and Nigeria) expressed stronger excitement about AI’s potential.

Existing research on organizational recruitment emphasizes the growing role of AI in interview processes (Lee and Rich, 2021; Nørskov et al., 2022; Suen et al., 2019; Sumathi et al., 2024). Park and Jung (2025) study highlights that job applicants often perceive AI-based interviews as less fair compared to traditional face-to-face interviews. Conducted across the USA and South Korea, their study found that American applicants, in particular, rated AI interviews lower in terms of job relevance, opportunity to perform, and two-way communication. These findings support the notion that the absence of human interaction in AI-based interviews can hinder the conveyance of nuanced information, thereby contributing to negative perceptions such as unfair evaluation (Chapman et al., 2003). While some studies have acknowledged the benefits of AI in recruitment, such as improved efficiency, consistency, and cost-effectiveness (Suen et al., 2019; Sumathi et al., 2024), the overall evidence remains inconclusive, especially when examining cross-cultural differences. For instance, perceptions of AI-driven interviews in contexts like China and Pakistan may diverge significantly from Western experiences, underscoring the need for more nuanced cultural analysis. Given these contradictions, the present study focuses on three key dimensions of applicant perception: perceived transparency, interactive communication, and job pursuit intention. These dimensions seek to clarify how cultural and contextual factors shape attitudes toward AI-based versus traditional human-based interview formats.

This study assumes AI-based interviews’ negative impact on job applicants’ attitudes and behaviors is less pronounced among Chinese individuals compared to Pakistani individuals. In China, widespread exposure to advanced technology and artificial intelligence in recent years has fostered greater trust in AI systems (Li et al., 2025; Qin et al., 2020), thereby reducing skepticism and negative perceptions. Chinese applicants demonstrate higher confidence in algorithmic decision-making and are more likely to trust machine-generated outcomes over human judgments (Min et al., 2024). In addition, empirical evidence suggests that psychological constructs, such as individual traits and social skills, play a significant role in helping candidates manage performance-related stress during job interviews (Speer et al., 2019). In the Chinese context, this coping ability appears to be enhanced when AI is perceived as an efficient and impartial interviewer (Zheng et al., 2024), reducing perceived biases and enhancing acceptance. In contrast, Pakistani applicants tend to exhibit higher levels of techno-skepticism and risk aversion, primarily due to lower levels of technological integration in their everyday lives (Khurshid et al., 2024). Previous studies have shown that Pakistani candidates express greater confidence in traditional human-led interviews and are more trusting of human-made final decisions (Nabi et al., 2015). As a result, they are more likely to view AI-generated interview questions and decision-making processes with suspicion, leading to heightened negative perceptions of the AI-based recruitment process (Park and Jung, 2025). These culturally driven differences suggest a divergence in how AI-based and human-based interviews are evaluated across national contexts. Accordingly, the following hypothesis is proposed:

*H1*: The negative impact of AI-based interviews, compared to human-based interviews, on perceived transparency is more pronounced among Pakistani applicants than among Chinese applicants.

### 2.2 AI-based interviews and interactive communication

Interactive communication refers to the extent to which job applicants have the opportunity to share their perspectives or provide input during the selection process (Acikgoz et al., 2020; Dineen et al., 2023), representing a key social dimension of perceived justice (Van Iddekinge et al., 2023). Cultural background significantly shapes how individuals value such interaction. In individualist societies, people often define themselves as distinct individuals, whereas in collectivist cultures, identity is closely tied to the group and interdependence (Komisarof and Akaliyski, 2025). This belief in individual uniqueness implies that selection methods lacking human interaction, such as AI-based interviews, may be perceived as inadequate for expressing or evaluating one’s personal qualities. In Western individualist contexts, prior research suggests that applicants see interactive communication during interviews as crucial for demonstrating their competencies (Park and Jung, 2025). When AI-based interviews restrict opportunities for social interaction, applicants may view these processes as less fair and less effective at capturing their unique attributes (Acikgoz et al., 2020; Min et al., 2024). In contrast, collectivist cultures, where group harmony and indirect communication are valued, may be less sensitive to the absence of human interaction, and applicants might show greater acceptance of impersonal selection methods (Köchling et al., 2025; Park and Jung, 2025).

Within the context of the distinctive cultural backgrounds of China and Pakistan (Yousaf et al., 2022), significant cultural differences may influence individuals’ responses to interactive communication in AI-based interviews. China, with its blend of collectivist traditions and growing technological familiarity (Yam et al., 2023), may foster a comparatively higher tolerance for AI-driven processes, with applicants potentially seeing less need for explicit human interaction during recruitment. Pakistani society, while also collectivist, is marked by strong preferences for interpersonal engagement and face-to-face communication in professional settings (Humaira Gul Saeed, 2016). This may result in Pakistani applicants placing greater value on interactive communication and perceiving its absence in AI-based interviews as a significant disadvantage.

In addition, China often exhibits a more fluid boundary between humans and technology, including a greater tendency to anthropomorphize AI and accept automated systems as socially competent (Lu et al., 2025). This may mitigate the perceived loss of interactive communication in AI-based recruitment. By contrast, in Pakistan, the novelty of AI in hiring and lower exposure to advanced recruitment technologies could amplify concerns over reduced interpersonal contact and hinder acceptance. Previous cross-cultural research has shown that justice perceptions linked to selection methods are more strongly associated with opportunities for direct interaction in some cultures than others (Chen and Yi, 2024). Therefore, we propose the following hypothesis:

*H2*. The negative impact of AI-based interviews, compared to human-based interviews, on perceived interactive communication is more pronounced among Pakistani applicants than among Chinese applicants.

### 2.3 AI-based interviews and job pursuit intention

Previous research has established that job pursuit intention, the willingness of applicants to continue pursuing employment opportunities after initial recruitment experiences, is a critical outcome of recruitment processes (Allen et al., 2022; Campion et al., 1997; Han et al., 2022). AI-driven recruitment technologies have notably reshaped these experiences by altering traditional recruitment methods (Suen et al., 2019). Park and Jung (2025) reported that while AI-based systems enhance operational efficiencies, accelerate selection cycles, and reduce hiring costs, applicant reactions to these technologies significantly vary across cultural contexts. Lee and Kim (2021a) found that applicant perceptions regarding AI-driven recruitment significantly influence organizational attractiveness and, consequently, applicants’ intention to apply. They argue that while advanced recruitment technologies can boost organizational branding in highly developed technological contexts, these same systems might adversely affect candidate perceptions and reduce job pursuit intentions in contexts less familiar or comfortable with AI.

Moreover, Park and Jung (2025) noted that applicant skepticism toward AI-driven recruitment intensifies in scenarios perceived as impersonal or lacking interpersonal engagement. Specifically, candidate reservations about emotional intelligence (Mantello et al., 2023), data privacy (Vimalkumar et al., 2021), and transparency (Kizilcec, 2016) in AI-driven processes, can undermine organizational attractiveness and discourage continued job pursuit. Such negative perceptions are particularly prominent among candidates who value interpersonal interactions in recruitment, a trait notably prevalent in less technologically integrated societies (Ejaz et al., 2026). Further, Kulal et al. (2025) and Park and Jung (2025) emphasized that AI-based recruitment can appear impersonal and potentially discriminatory in regions unfamiliar with technological innovations. Thus, candidates from less developed technological contexts are likely to exhibit reduced trust and acceptance toward AI-based recruitment systems, which in turn negatively influences their intention to pursue employment opportunities further.

Applying these insights to the specific cultural comparison between China and Pakistan, it is logical to assume differences in applicants’ responses to AI-based interviews. China’s relative technological advancement (Li et al., 2025), extensive familiarity with AI tools (Zheng et al., 2024), and higher societal trust in automated systems may buffer applicants from negative reactions (Choung et al., 2023), thereby sustaining their job pursuit intentions despite reduced interpersonal contact during recruitment. In contrast, applicants from Pakistan, where direct interpersonal interactions in professional settings are highly valued, and exposure to AI-driven selection tools remains limited (Bibi, 2019), are more likely to perceive AI-based interviews negatively. This skepticism can manifest in diminished trust and reduced willingness to pursue employment opportunities with organizations utilizing AI-based recruitment processes. Consequently, we hypothesize:

H3: The negative impact of AI-based interviews, compared to human-based interviews, on job pursuit intention will be more pronounced among Pakistani applicants than among Chinese applicants.

### 2.4 Moderating role of trust in AI systems

AI is increasingly utilized in human resource practices, transforming traditional recruitment methods by introducing automation, predictive analytics, and data-driven decision-making processes (Malik et al., 2023b; Tambe et al., 2019). Questions regarding transparency, fairness, and ethical implications of these processes have emerged prominently in recent research (Hunkenschroer and Luetge, 2022; Zuiderveen Borgesius, 2020). Although AI systems are increasingly recognized for streamlining recruitment and mitigating biases inherent in human judgment (Chamorro-Premuzic et al., 2019), candidate trust in AI-driven systems remains an important issue influencing applicant reactions (Basch et al., 2020; Lacroux and Martin-Lacroux, 2022). Glikson and Woolley (2020) highlighted that trust is a critical determinant of recruiters’ and candidates’ acceptance of AI systems in resume screening, underscoring that individuals tend to exhibit skepticism towards automated recommendations compared to human ones, especially when decisions involve subjective evaluation or significant personal outcomes.

Although prior technology acceptance research has commonly positioned trust as an antecedent of behavioral intention or as a mediator linking construct to user outcomes (Choung et al., 2023; Zhang et al., 2021), the present study considered trust as a moderator because of the theoretical logic and design of this research. In AI-based recruitment, interview type functions as an external signal that may shape candidates’ perceptions of transparency, fairness, and procedural acceptability (Zhang et al., 2025). However, the meaning and strength of this signal are unlikely to be identical for all candidates. Applicants with higher trust in AI systems may interpret AI-based interviews as more objective, consistent, and acceptable (Suen and Hung, 2023), whereas applicants with lower trust may interpret the same AI-based interview format as less transparent, more uncertain, or less fair. Thus, trust does not necessarily transmit the effect of interview type; rather, it conditions how strongly interview type is associated with applicant perceptions. This argument is consistent with Fairness Heuristic Theory, which suggests that individuals rely on general trust-related judgments when evaluating procedures under uncertainty (Lind, 2001). It also aligns with contingency-based accounts of signaling, in which the effect of a signal depends on the receiver’s prior disposition toward the signal source (Bangerter et al., 2012). In the present design, trust in AI is conceptualized as a relatively stable pre-existing belief that candidates bring into the recruitment encounter, rather than as an attitude produced by a single interview experience.

At the same time, trust in technology should not be treated as a purely individual-level disposition detached from cultural context. Trust may be shaped by broader cultural and institutional conditions, including generalized trust, confidence in systems and authorities, uncertainty avoidance, and expectations regarding interpersonal versus impersonal decision-making (Gillespie et al., 2023). In collectivistic contexts, trust may also be influenced by whether a system is perceived as legitimate, institutionally endorsed, and aligned with accepted social or organizational norms. Because China and Pakistan differ in technological exposure, institutional context, and uncertainty avoidance (Alam et al., 2024), their baseline levels of trust in AI may differ. Therefore, part of the cross-cultural difference in applicant reactions may operate through culturally shaped trust.

However, the present study distinguishes between differences in the baseline level of trust and the moderating function of trust. Applicants who hold stronger trust in AI are more likely to interpret automated interview procedures as reliable, consistent, and acceptable. By contrast, applicants with lower trust may be more likely to view the same procedures as uncertain, opaque, or less fair. Thus, although cultural context may influence how much trust applicants initially place in AI systems, trust is expected to condition the relationship between AI-based interviews and applicant perceptions across both samples.

Research indicates that trust moderates the acceptance of AI recommendations in HR contexts, significantly shaping perceptions of transparency (Suen and Hung, 2023). Specifically, higher trust levels in AI systems lead candidates to perceive AI-driven processes as fairer and more transparent, increasing acceptance and reducing resistance (Gonzalez et al., 2022). Conversely, diminished trust can amplify concerns about the opacity and impartiality of AI systems, negatively influencing perceived transparency (Ananny and Crawford, 2018; Kizilcec, 2016).

Cultural background significantly shapes how candidates interpret and respond to AI-driven recruitment, with notable variations between technological adoption levels across different societies. Previous studies suggested that in countries where digital technologies are deeply integrated into daily life, applicants may approach AI tools with greater familiarity and confidence (Kitchin, 2017; Kshetri, 2020; Westjohn et al., 2022). In contrast, cultures that place a stronger value on human interaction or have limited exposure to AI may foster more critical attitudes, leading to reduced trust and lower perceived transparency in automated hiring processes (Chen et al., 2013). For instance, Chinese candidates may inherently hold more positive views of AI systems due to greater technological exposure, whereas Pakistani candidates may favor human interaction during interviews, resulting in initially more skeptical perceptions of AI-based processes.

Nonetheless, recent studies argue for certain consistent patterns, particularly concerning trust’s moderating role in transparency perceptions. Van Esch et al. (2019) found that the performance expectancy of AI consistently predicts ethical perceptions and trust, implying potential cross-cultural similarity. This suggests that while baseline trust levels in AI may differ culturally, the fundamental psychological mechanism through which trust operates may extend across cultural boundaries. Therefore, despite differences in baseline perceptions, trust’s influence on how interview type shapes transparency perception is anticipated to follow a similar moderating pattern in both cultural settings. Based on this integration of the existing literature, we propose the following hypothesis:

*H4*: Trust in the AI system moderates the relationship between interview type (AI-based vs. human-based) and perceived transparency, and this moderating effect is comparable across cultural contexts (China and Pakistan).

Building upon the established role of trust in shaping transparency perceptions, existing literature on human psychology has further validated trust’s critical role in determining behavioral responses toward interactive communication. Individuals who demonstrate greater trust in AI-driven systems generally perceive automated interactions as more credible and satisfying substitutes for human-based interactions (Chi et al., 2020). This perceived adequacy of interaction in AI-based interviews stems from applicants’ belief in the reliability, fairness, and accuracy of algorithmic assessments, ultimately fostering positive recruitment experiences despite reduced human interaction (Glikson and Woolley, 2020). Woods et al. (2020) reported that individuals who understand and trust AI systems typically experience fewer negative reactions towards automated processes. Conversely, insufficient trust amplifies concerns regarding the fairness, consequently heightening perceptions that AI-driven selection lacks meaningful interpersonal engagement (Gessl et al., 2019).

Although the cultural foundations of trust may differ across societies, the psychological role of trust in reducing negative reactions to automated interaction may be comparable across the two samples examined. Therefore, we hypothesize: H5: Trust in the AI system moderates the relationship between interview type (AI-based vs. human-based) and interactive communication, and this moderating effect is comparable across cultural contexts (China and Pakistan).

Extending this trust framework to job pursuit intentions, several scholars have noted that criticisms of AI-driven recruitment processes often intensify candidates’ psychological concerns, which in turn adversely affect their perceptions and intentions to apply (Fernández-Martínez and Fernández, 2020; Kammerer, 2021). Trust in AI systems emerges as a pivotal factor capable of mitigating these negative perceptions and converting them into positive behavioral intentions. For example, Arora and Mittal (2024) demonstrated a positive indirect effect of trust in AI task management, while Marimon et al. (2025) emphasized the critical role of trust in the relationship between using AI tools and individual work behavior. Given the established pattern that trust mechanisms operate similarly across cultural contexts for both transparency and interactive communication, we expect this consistency to extend to job pursuit intentions. Based on the above logical synthesis of existing literature, this study hypothesizes:

H6: Trust in the AI system moderates the relationship between interview type (AI-based vs. human-based) and job pursuit intention, and this moderating effect is comparable across cultural contexts (China and Pakistan).

Based on the above discussion, from a cultural perspective, we can hypothesize that:

*H7*: The effects of AI-based interviews, compared to human-based interviews, on perceived transparency, interactive communication, and job pursuit intention differ between Chinese and Pakistani applicants, whereas the moderating influence of trust in the AI system is comparable across both cultural contexts.

**Figure 1:**
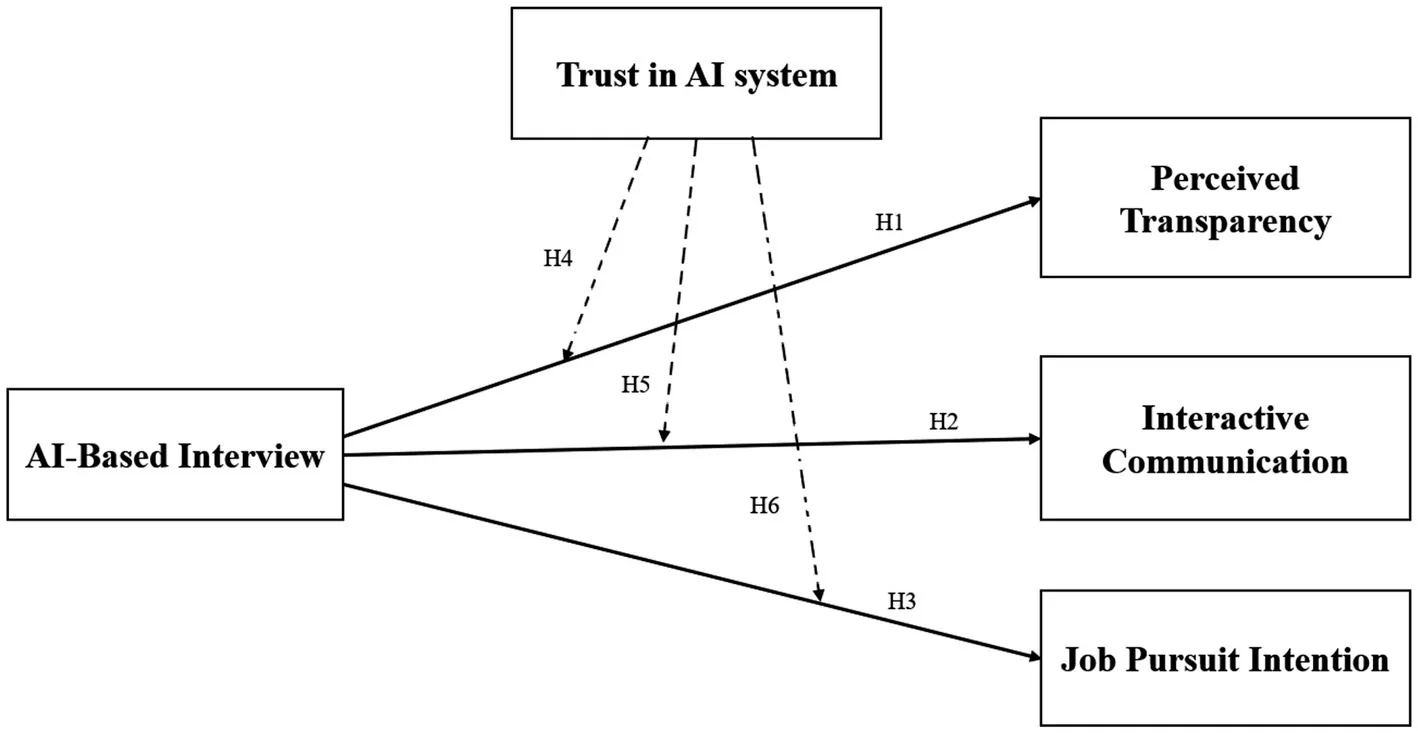
Conceptual framework.

Figure 1 represents the conceptual framework used for this study.

## 3 Research methodology

### 3.1 Data sample

To empirically examine the proposed research hypotheses, a cross-sectional survey was conducted across two countries. Data analysis was performed using Partial Least Squares Structural Equation Modeling (PLS-SEM) via SmartPLS 4.0. PLS-SEM is a robust analytical technique widely recognized for its efficacy in estimating complex causal relationships within management research frameworks (Gudergan et al., 2008). The study employed a convenience sampling approach for data collection. The target population for this study consisted of job seekers, including both recent graduates and currently employed individuals seeking better career opportuniti12twelve months. A mandatory screening question (“Have you completed a job interview in which an AI system, not a human recruiter, evaluated your answers?”) ensured that only AI-experienced respondents proceeded to the main questionnaire. Data were collected from July 2025 to August 2025, through both in-person distribution and online distribution. In each country, a total of 250 questionnaires were distributed across multiple cities to ensure a broad and regionally diverse sample. In Pakistan, data collection was conducted in Islamabad, Lahore, Faisalabad, and Peshawar. A total of 224 were returned, yielding a response rate of 89.6%. After removing 11 questionnaires due to incomplete or missing data, 213 responses were retained for further analysis. The demographic distribution of the Pakistani sample indicated that 128 respondents (60.1%) were male, while 85 respondents (39.9%) were female. In China, data were collected from four major cities, including Beijing, Shanghai, Nanjing, and Wuhan. A total of 239 completed questionnaires were received, representing a response rate of 95.6%. 14 questionnaires with missing data were excluded, resulting in a final sample of 225 usable responses. Among the Chinese participants, 119 (52.9%) were female, and 106 (47.1%) were male. The adequacy of the sample size was assessed according to the rule of thumb for PLS-SEM, which recommends a minimum sample size of ten times the maximum number of structural paths directed at any latent variable (Hair et al., 2014). In the present model, six arrows point toward endogenous constructs, requiring a minimum of 60 valid cases. Accordingly, the final sample sizes in both Pakistan (*n* = 213) and China (*n* = 225) substantially exceed this threshold, supporting the suitability of the dataset for PLS-SEM analysis.

In Pakistan, the questionnaires were distributed in English (official language). While in China, the questionnaires were provided in both English and Chinese versions. A professional language expert initially translated the Chinese-language version and subsequently validated it by a senior academic expert in Business and Management. The questionnaire comprised two main sections. The first section included demographic information, including respondents’ country, age, gender, and educational qualifications (see Table 1). The second section consisted of items designed to measure the variables under investigation, using a five-point Likert scale that ranged from 1 (strongly disagree) to 5 (strongly agree).

**Table 1:** Demographic characteristics.

Characteristic	Category	Frequency (n)	Percentage (%)
China data sample			
Gender	Male	106	47.1%
	Female	119	52.9%
City	Beijing	57	25.3%
	Shanghai	59	26.2%
	Nanjing	56	24.9%
	Wuhan	53	23.6%
Age	Under 25 Years	48	21.3%
	26–35 Years	96	42.7%
	36–45 Years	56	24.9%
	Above 45 Years	25	11.1%
Education	Bachelor’s Degree	103	45.8%
	Master’s Degree	110	48.9%
	Doctorate/PhD	12	5.3%
Pakistan data sample			
Gender	Male	128	60.1%
	Female	85	39.9%
City	Islamabad	58	27.2%
	Lahore	52	24.4%
	Faisalabad	54	25.4%
	Peshawar	49	23.0%
Age	Under 25 Years	54	25.4%
	26–35 Years	87	40.8%
	36–45 Years	44	20.7%
	Above 45 Years	28	13.1%
Education	Bachelor’s Degree	79	37.1%
	Master’s Degree	127	59.6%
	Doctorate/PhD	07	3.3%

### 3.2 Operationalization and analysis of interview type

Interview type was operationalized based on respondents’ actual prior experience with AI-based interviews rather than through a between-subjects experimental manipulation. All respondents included in the final analysis had completed at least one AI-based video interview within the previous 12 months, as confirmed by the mandatory screening question. Therefore, interview type was not coded as a binary experimental variable.

The independent variable, labelled “AI-Based Interview,” was measured as a reflective latent construct using ten items adapted from prior validated scales. This construct captured applicants’ perceived experience of the AI-based interview process. The traditional human-based interview was used as a conceptual comparison reference, allowing respondents to evaluate AI-based interviews in relation to conventional human-led interview expectations. No participant was assigned to a traditional human-based interview condition, nor was any participant asked to evaluate AI-based interview items after receiving a human-based interview description.

Accordingly, the structural model was based on respondents’ subjective perceptions of AI-based interviews, not on experimental group membership. Since no experimental condition was manipulated, a manipulation check was not applicable. The screening question served as an eligibility and experience check to ensure that all retained respondents had genuine and recent AI-interview experience. The two samples, China (*n* = 225) and Pakistan (*n* = 213), were analyzed separately and then compared using multi-group analysis.

After providing informed consent, respondents first answered the screening question to confirm whether they had completed an AI-based video interview during the previous 12 months. Respondents who answered “No” were not included in the final dataset. Eligible respondents were then instructed to recall their most recent AI-based video interview experience and to answer all subsequent items with reference to that experience. The questionnaire then measured perceptions of the AI-based interview process, perceived transparency, interactive communication, job pursuit intention, trust in AI, and demographic information.

### 3.3 Measurement

AI-Based Interviews: The AI-based interview construct refers to applicants’ perceived experience of interviews conducted through AI-enabled systems, in which the AI system evaluated candidate responses without direct human recruiter interaction during the interview stage. All analyzed respondents had actual recent experience with AI-based interviews and answered the measurement items with reference to that experience. The construct was measured as a reflective latent variable rather than as an experimentally assigned condition. The traditional human-based interview was referenced only as a conceptual comparison point representing conventional interviews conducted by human recruiters. The scale items were adapted from prior validated instruments developed by Höddinghaus et al. (2021) and Wongras and Tanantong (2023). A total of 10 items were included, such as “I believe that the AI system fairly evaluates my capability during the interview process.

Perceived Transparency: This construct was measured using six items adapted from Wongras and Tanantong (2023), focusing on candidates’ perceptions of fairness, clarity of evaluation criteria, and openness throughout the interview process. The items were modified to capture transparency in AI-based interview formats. E.g., “The AI interview process is transparent about the factors that are considered in evaluating candidates.”

Interactive Communication: Seven items were adapted from Ramos-Morcillo et al. (2020) to measure interactive communication. The existing scales were modified to the study context, and the items assess bidirectional communication quality and responsiveness, examining whether AI-based formats can maintain meaningful dialogue comparable to human interactions. E.g., “I was given the opportunity to actively express myself during the interview process.”

Job Pursuit Intention: To evaluate applicants’ continued interest in the organization following the interview experience, seven items were adapted from Xiang H. et al. (2023) and Tanantong and Wongras (2024). These items measure behavioral intentions regarding job pursuit, organizational attractiveness, and recommendation behaviors as influenced by the interview process quality. E.g., “I am still interested in pursuing a job with this organization after the interview experience.”

Trust in AI System: Trust in the AI system was measured using six items adapted from Choung et al. (2023), reflecting confidence in the AI’s capability to make fair, unbiased, and reliable recruitment decisions. The scale captures multidimensional trust aspects, including competence-based trust, benevolence-based trust, and integrity-based trust, specific to algorithmic decision-making contexts. E.g., “I trust that the AI system will make fair hiring decisions.

Control variables: The demographic variables, including age, gender, academic qualification, and work experience, were used as control variables in this study. According to Ye et al. (2022), control variables possibly show their influence on the targeted variables of this study.

## 4 Data analysis and results

### 4.1 Common method bias (CMB)

CMB should be assessed when data are collected through self-administered questionnaires, specifically, when the independent variables and dependent variables are gathered from the same person (Podsakoff et al., 2003). Therefore, several procedural and statistical steps were implemented to address this concern. First, to reduce consistency artifacts and response bias, the questionnaire was organized into clearly separated sections. Each section was introduced with brief framing instructions so that the predictor and outcome measures were psychologically separated rather than presented as a single continuous block of items. Second, trained data collectors assured participants of the confidentiality and anonymity of their responses, encouraging honest answers. Finally, Harman’s single-factor test was conducted to statistically assess the presence of CMB. The analysis showed that a single factor accounted for only 38.273% of the total variance, which is below the commonly accepted threshold of 50%. In addition, following Kock (2015), full collinearity VIF values were examined. Kock recommends that VIF values should not exceed 3.3. The obtained VIF values in both samples ranged from 1.386 to 3.185 after consistent PLS algorithm calculations, indicating that CMB is not a serious concern in this study. Together, these procedural and statistical results suggest that common method bias is unlikely to explain the observed relationships among the study variables.

### 4.2 Reliability and validity

**Figure 2:**
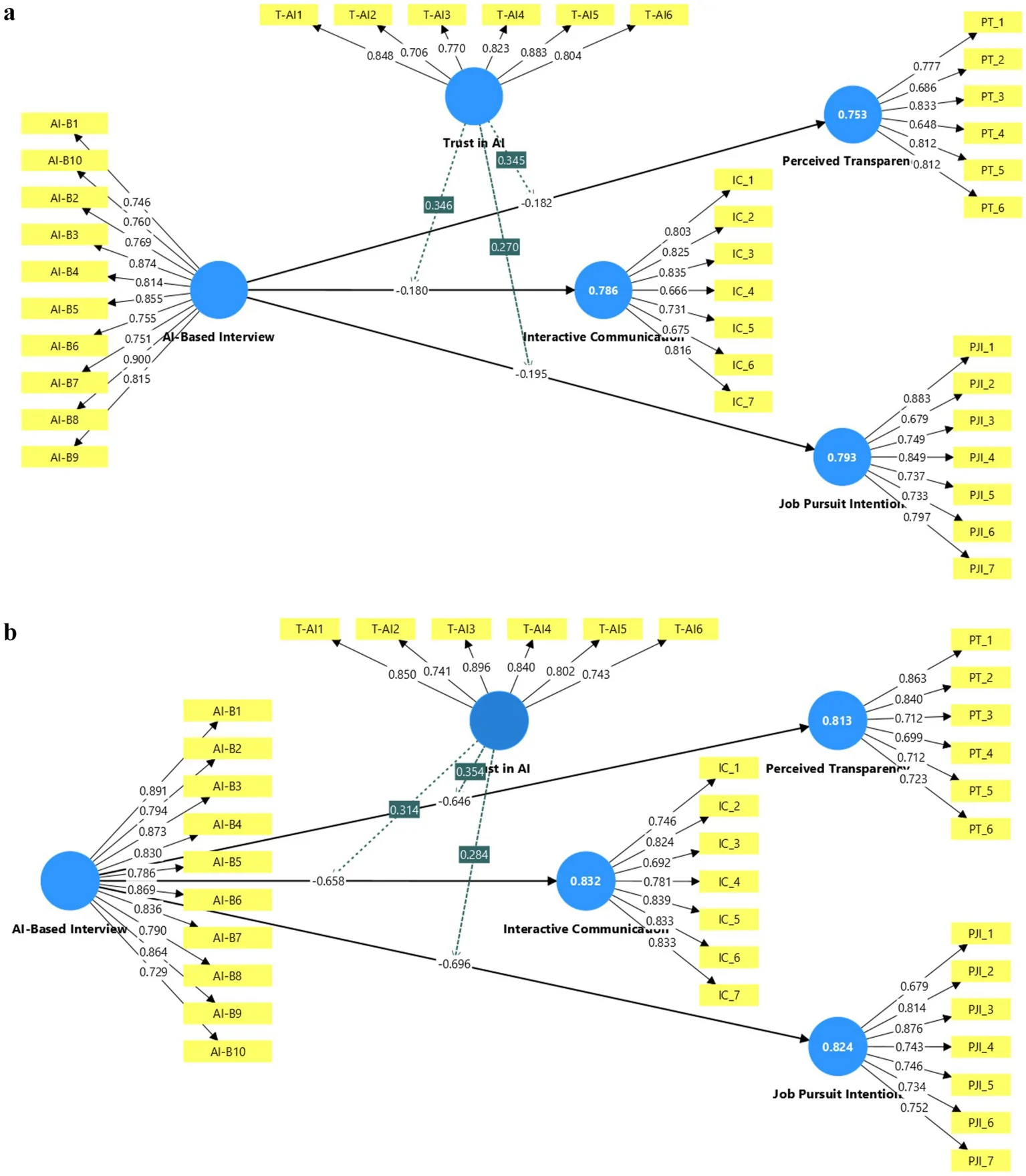
Measurement model. **(a)** China. **(b)**. Pakistan.

The first step involved evaluating the measurement model for convergent validity. This was determined by examining outer loadings, Composite Reliability (CR), and Average Variance Extracted (AVE). As reported in Table 2, all item loadings were above the suggested threshold of 0.60 (see Figures 2a,b) (Hair et al., 2021). In addition, the composite reliability and Cronbach’s alpha values for all constructs surpassed the recommended criteria of 0.70, indicating strong internal consistency. The AVE values, which represent the overall variance in the indicators accounted for by variables, meet the recommended value of 0.50 (Hair et al., 2021), further confirming convergent validity.

**Table 2:** Validity and reliability.

Construct/Item	λ	Cronbach’s Alpha	CR	AVE	VIF
China data sample					
AI-based interview		0.940	0.948	0.649	
AI-B1	0.746				2.029
AI-B2	0.769				2.011
AI-B3	0.874				2.174
AI-B4	0.814				3.147
AI-B5	0.855				2.642
AI-B6	0.755				2.967
AI-B7	0.751				2.022
AI-B8	0.900				2.037
AI-B9	0.815				3.080
AI-B10	0.760				2.573
Perceived transparency		0.856	0.893	0.585	
PT-1	0.777				2.038
PT-2	0.686				1.830
PT-3	0.833				1.470
PT-4	0.648				2.120
PT-5	0.812				1.386
PT-6	0.812				2.074
Interactive communication		0.882	0.909	0.589	
IC-1	0.803				2.174
IC-2	0.825				2.284
IC-3	0.835				2.437
IC-4	0.666				1.541
IC-5	0.731				1.711
IC-6	0.675				1.532
IC-7	0.816				2.239
Job pursuit intention		0.890	0.914	0.605	
PJI-1	0.883				3.083
PJI-2	0.679				1.590
PJI-3	0.749				1.801
PJI-4	0.849				2.523
PJI-5	0.737				1.759
PJI-6	0.733				1.731
PJI-7	0.797				2.078
Trust in the AI system		0.892	0.918	0.652	
T-AI1	0.848				2.543
T-AI2	0.706				1.592
T-AI3	0.770				1.833
T-AI4	0.823				2.278
T-AI5	0.883				3.010
T-AI6	0.804				2.006
Pakistan data sample					
AI-based interview		0.948	0.956	0.685	
AI-B1	0.891				3.081
AI-B2	0.794				1.928
AI-B3	0.873				2.355
AI-B4	0.830				3.119
AI-B5	0.786				2.767
AI-B6	0.869				2.277
AI-B7	0.836				3.110
AI-B8	0.790				2.879
AI-B9	0.864				2.354
AI-B10	0.729				3.027
Perceived transparency		0.853	0.891	0.579	
PT-1	0.863				2.532
PT-2	0.840				2.314
PT-3	0.712				1.589
PT-4	0.699				1.520
PT-5	0.712				1.535
PT-6	0.723				1.632
Interactive communication		0.902	0.923	0.631	
IC-1	0.746				1.841
IC-2	0.824				2.248
IC-3	0.692				1.565
IC-4	0.781				1.972
IC-5	0.839				2.495
IC-6	0.833				2.368
IC-7	0.833				2.412
Job pursuit intention		0.881	0.908	0.586	
PJI-1	0.679				1.497
PJI-2	0.814				2.154
PJI-3	0.876				2.872
PJI-4	0.743				1.782
PJI-5	0.746				1.716
PJI-6	0.734				1.732
PJI-7	0.752				1.815
Trust in the AI system		0.897	0.921	0.663	
T-AI1	0.850				2.578
T-AI2	0.741				1.716
T-AI3	0.896				3.185
T-AI4	0.840				2.370
T-AI5	0.802				2.069
T-AI6	0.743				1.813

The next phase involved evaluating discriminant validity, which ensures that each construct is distinct from others and not simply measuring the same underlying concept. This is typically indicated by low correlations between the target construct and other variables. As shown in Table 3, the square root of the AVE for each construct (represented by the diagonal values) exceeds its correlations with other constructs. This finding supports the adequacy of discriminant validity, in line with the criteria established by Fornell and Larcker (1981).

**Table 3:** Discriminant validity (Fornell and Larcker).

Construct	AI-based interview	Interactive communication	Job pursuit intention	Perceived transparency	Trust in AI
China					
AI-based interview	0.806				
Interactive communication	−0.251	0.767			
Job pursuit intention	−0.257	0.691	0.778		
Perceived transparency	−0.252	0.567	0.659	0.765	
Trust in AI	−0.031	0.681	0.514	0.459	0.808
Pakistan					
AI-based interview	0.828				
Interactive communication	−0.666	0.794			
Job pursuit intention	−0.702	0.674	0.766		
Perceived transparency	−0.663	0.584	0.584	0.761	
Trust in AI	0.065	0.486	0.447	0.443	0.814

Recent studies have raised concerns about the Fornell and Larcker (1981) criterion, indicating that it may not consistently identify issues with discriminant validity in many research contexts (Henseler et al., 2015). To address this, Henseler et al. (2015) proposed the heterotrait-monotrait (HTMT) ratio of correlations, which is grounded in the multitrait-multimethod approach, as a more reliable alternative for assessing discriminant validity. In this study, discriminant validity was further evaluated using the HTMT method (see Table 4). According to Henseler et al. (2015), the HTMT value exceeding the 0.85 threshold suggests potential discriminant validity issues. However, all HTMT values in this analysis were below 0.85, confirming that discriminant validity was satisfactorily established.

**Table 4:** Discriminant validity (HTMT).

Construct	AI-based interview	Interactive communication	Job pursuit intention	Perceived transparency	Trust in AI
China					
AI-based interview					
Interactive communication	0.267				
Job pursuit intention	0.269	0.652			
Perceived transparency	0.275	0.598	0.601		
Trust in AI	0.067	0.471	0.401	0.663	
Pakistan					
AI-based interview					
Interactive communication	0.714				
Job pursuit intention	0.762	0.717			
Perceived transparency	0.732	0.604	0.539		
Trust in AI	0.099	0.535	0.495	0.495	

### 4.3 Model fit

Model fit assesses the extent to which a conceptual model represents real data. In PLS-SEM, the SRMR, NFI, and RMS_theta are commonly used fit indices to evaluate the overall adequacy of the model. A SRMR value below 0.08 indicates a good fit, signifying close correspondence between the model and empirical data (Hair et al., 2021). The reported SRMR of both data samples is below the 0.08 threshold, as given in Table 5, indicating strong alignment between the model-suggested and actual data correlations. Thus, the model effectively reflects the exact underlying relationships within the dataset. These indices provide important information regarding the suitability and quality of the model, as summarized in Table 5.

**Table 5:** Model fitness.

Model fit index	Saturated model	Estimated model
China		
SRMR	0.053	0.061
d_ULS	1.119	2.064
d_G	0.491	0.595
Chi-square	1489.405	1542.850
NFI	0.931	0.873
Pakistan		
SRMR	0.058	0.069
d_ULS	1.146	2.174
d_G	0.510	0.603
Chi-square	1588.731	1620.912
NFI	0.952	0.889

### 4.4 Structural model

In this step, the hypothesized relationships among the study variables were examined. First, the direct relationships were analyzed separately for the Chinese and Pakistani samples.

The results for the Chinese sample show that perceived AI-based interview experience was negatively and significantly associated with perceived transparency (*β* = −0.182, t = 4.149, *p* < 0.001), interactive communication (*β* = −0.180, t = 4.524, *p* < 0.001), and job pursuit intention (*β* = −0.195, t = 5.082, p < 0.001) (see Table 6). Similarly, the results for the Pakistani sample show that perceived AI-based interview experience was negatively and significantly associated with perceived transparency (*β* = −0.646, t = 13.708, *p* < 0.001), interactive communication (*β* = −0.658, t = 15.268, *p* < 0.001), and job pursuit intention (*β* = −0.696, t = 16.555, *p* < 0.001). These findings indicate that the negative associations were stronger in magnitude in the Pakistani sample than in the Chinese sample for perceived transparency, interactive communication, and job pursuit intention. Consequently, H1, H2, and H3 were supported.

**Table 6:** Structural estimates (hypothesis testing).

Relationships	β	S.D.	T-value	*P*-value	Decision	F^2^
China						
AI-based interview ➔ perceived transparency	−0.182	0.044	4.149	0.000	Accepted	0.131
AI-based interview ➔ interactive communication	−0.180	0.040	4.524	0.000	Accepted	0.149
AI-based interview ➔ job pursuit intention	−0.195	0.038	5.082	0.000	Accepted	0.181
Pakistan						
AI-based interview ➔ perceived transparency	−0.646	0.047	13.708	0.000	Accepted	0.134
AI-based interview ➔ interactive communication	−0.658	0.043	15.268	0.000	Accepted	0.139
AI-based interview ➔ job pursuit intention	−0.696	0.042	16.555	0.000	Accepted	0.147

**Figure 3:**
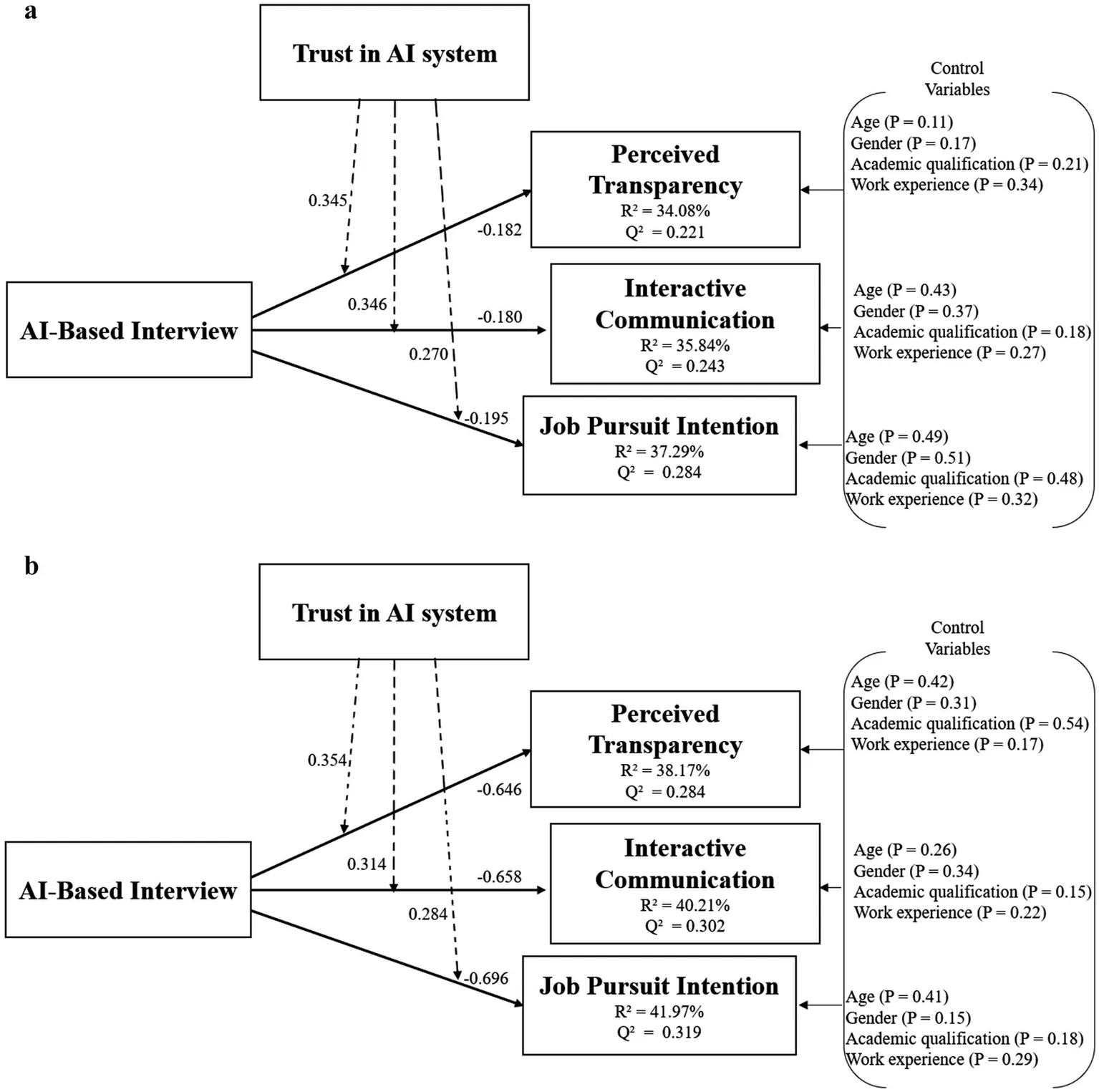
Structural analysis. **(a)** China. **(b)**. Pakistan.

Notably, the results of all control variables are insignificant and have no impact on the study’s main variables (see Table 6). Furthermore, in the Chinese data sample, the AI-based interviews account for 34.8% of the variance in perceived transparency (R^2^ = 0.348), 35.8% in Interactive Communication (R^2^ = 0.358), and 37.2% in Job pursuit intention (R^2^ = 0.372). In the Pakistani data sample, the AI-based interviews explain 38.17% of the variance in perceived transparency (R^2^ = 0.381), 40.2% in Interactive Communication (R^2^ = 0.402), and 41.9% in Job pursuit intention (R^2^ = 0.419), as given in Figures 3a,b. Overall R^2^ values exceed the threshold of 0.26 recommended by Cohen (2013), signifying that the model possesses substantial explanatory power. Moreover, the effect sizes (f^2^) provide a more comprehensive understanding of the model’s explanatory power. Therefore, f^2^ was calculated by examining the change in R^2^ values when a specific exogenous construct is omitted from the model (Hair et al., 2013). The f^2^ value of 0.02 is considered a small, 0.15 a medium, and 0.35 a large effect (Cohen, 2013). As reported in Table 6, all observed relationships demonstrated medium effect sizes, indicating meaningful contributions to the endogenous constructs. After reporting R^2^ and f2, the predictive relevance (Q^2^) was also reported by using the predictive sample reuse technique, as recommended by Chin et al. (2008). A Q^2^ value greater than zero indicates that the model has predictive relevance, whereas a value below zero implies insufficient predictive relevance. The Q^2^ values demonstrated adequate predictive relevance, as shown in Figures 3a,b.

### 4.5 Moderation analysis

In the moderation analysis, we assess the moderating effect of applicants’ trust in the AI system. To empirically test this moderating effect, the Partial Least Squares product-indicator approach was employed. This method was selected based on its demonstrated capacity to yield more precise assessments of moderation effects (Henseler and Fassott, 2010). The results for both samples revealed positive and significant interaction effects between trust in AI and perceived AI-based interview experience. In the Chinese sample, trust in AI significantly moderated the relationships between AI-based interviews and perceived transparency (*β* = 0.345, t = 10.240, *p* < 0.001), interactive communication (*β* = 0.346, t = 10.858, *p* < 0.001), and job pursuit intention (*β* = 0.270, t = 8.300, *p* < 0.001). Similarly, in the Pakistani sample, trust in AI significantly moderated the relationships between AI-based interviews and perceived transparency (*β* = 0.354, t = 10.188, *p* < 0.001), interactive communication (*β* = 0.314, t = 9.143, *p* < 0.001), and job pursuit intention (*β* = 0.284, t = 9.580, *p* < 0.001), as reported in Table 7.

**Table 7:** Moderation analysis.

Relationships	β	S.D.	*T*-value	*P*-value	Decision	F^2^
China						
Trust in AI x AI-based interview ➔ perceived transparency	0.345	0.034	10.240	0.000	Accepted	0.141
Trust in AI x AI-based interview ➔ interactive communication	0.346	0.032	10.858	0.000	Accepted	0.143
Trust in AI x AI-based interview ➔ job pursuit intention	0.270	0.033	8.300	0.000	Accepted	0.138
Pakistan						
Trust in AI x AI-based interview ➔ perceived transparency	0.354	0.035	10.188	0.000	Accepted	0.144
Trust in AI x AI-based interview➔ interactive communication	0.314	0.034	9.143	0.000	Accepted	0.129
Trust in AI x AI-based interview ➔ job pursuit intention	0.284	0.030	9.580	0.000	Accepted	0.132

**Figure 4:**
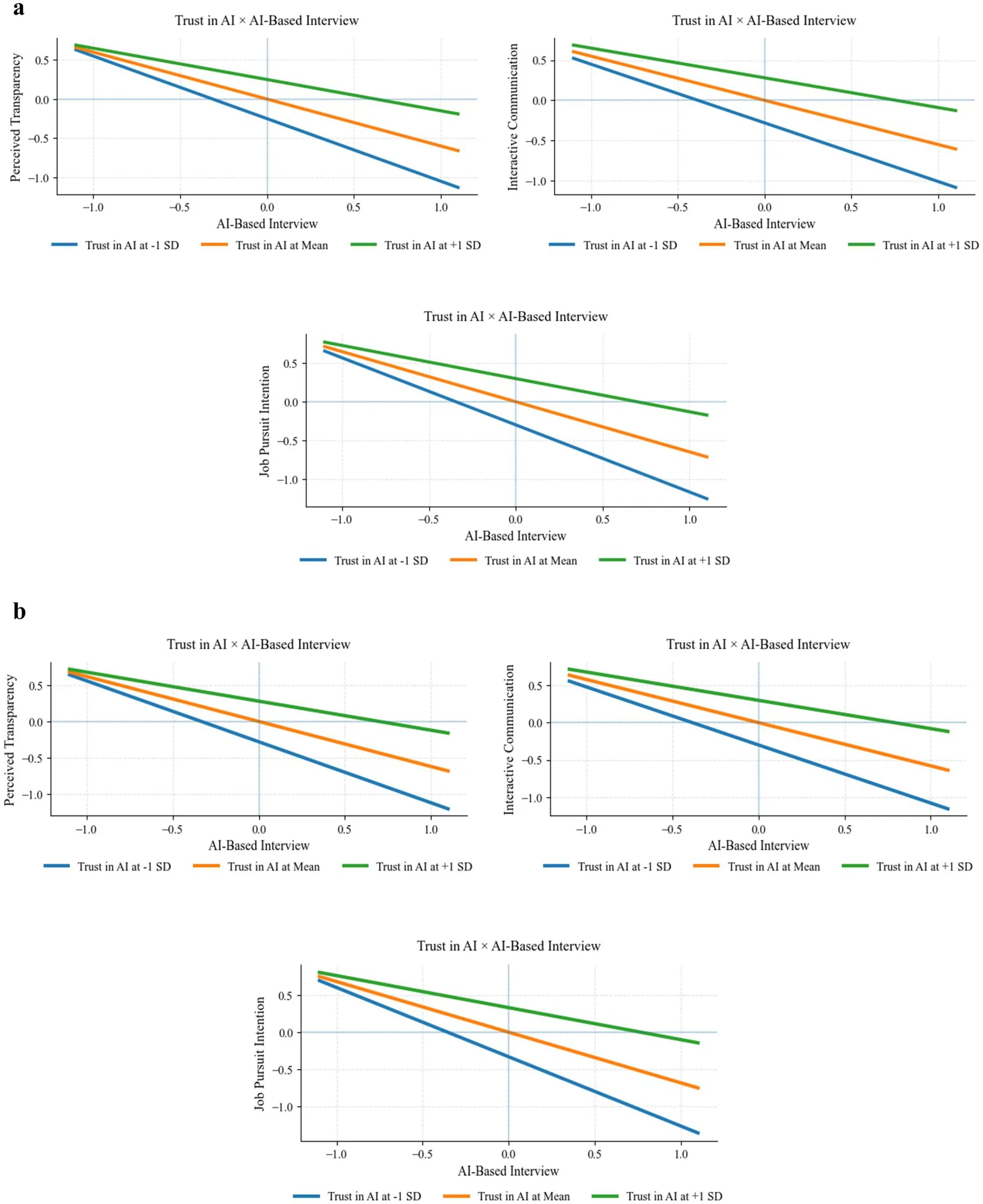
Moderation analysis. **(a)** China. **(b)**. Pakistan.

The positive interaction coefficients should be interpreted in relation to the negative direct relationships between AI-based interviews and applicant outcomes. Specifically, the positive moderation coefficients indicate that trust in AI attenuates the negative associations between AI-based interviews and perceived transparency, interactive communication, and job pursuit intention. Thus, applicants with higher levels of trust in AI reported less unfavorable evaluations of AI-based interviews than applicants with lower levels of trust. These findings provide support for H4, H5, and H6 by demonstrating that trust in AI significantly moderates the relationships between AI-based interviews and all three applicant outcomes in both samples. The cross-sample comparability of these moderation effects is examined further through the multi-group analysis reported in Section 4.7 (Figure 4).

### 4.6 Measurement invariance assessment

Before conducting the multi-group analysis, measurement invariance was assessed using the measurement invariance of composite models (MICOM) procedure in SmartPLS. This assessment was necessary because the study compares structural relationships between the Chinese and Pakistani samples. Establishing measurement invariance ensures that observed group differences are not caused by differences in how respondents interpret the measurement items.

The MICOM procedure was conducted in three steps. First, configural invariance was established because the same constructs, indicators, data treatment procedures, algorithm settings, and model specifications were applied to both country samples. Second, compositional invariance was examined through permutation testing. As shown in Table 8, the original correlation values for all constructs were equal to or greater than the 5% quantile values, and all permutation *p*-values were greater than 0.05. Therefore, compositional invariance was established for all constructs.

**Table 8:** Measurement invariance assessment using MICOM.

Construct	Original correlation	5% quantile	Step 2 *p*-value	Compositional invariance	Mean equality *p*-value	Variance equality p-value	MICOM conclusion
Interactive communication	1.000	0.999	0.721	Yes	0.908	0.879	Full invariance
AI-Based interview	1.000	1.000	0.086	Yes	0.917	0.901	Full invariance
Job pursuit intention	1.000	0.999	0.299	Yes	0.902	0.969	Full invariance
Perceived transparency	1.000	0.998	0.870	Yes	0.915	0.970	Full invariance
Trust in AI	1.000	0.999	0.448	Yes	0.927	0.767	Full invariance

Third, equality of composite means and variances was assessed. The results showed that the original mean differences for all constructs fell within the corresponding 95% confidence intervals, and all permutation p-values were greater than 0.05. Similarly, the original variance differences also fell within the corresponding 95% confidence intervals, with all permutation p-values greater than 0.05. Therefore, equality of composite means and variances was established.

### 4.7 Multi-group analysis

After establishing measurement invariance through the MICOM procedure, multi-group analysis was conducted to examine whether the structural relationships differed between the Chinese and Pakistani samples. This step was necessary to determine whether the relationships between perceived AI-based interview experience and applicant outcomes varied across the two cultural contexts.

The analysis revealed significant differences in the paths from AI-based interviews to perceived transparency, interactive communication, and job pursuit intention. Specifically, the path differences were significant for perceived transparency (path difference = 0.464, *p* = 0.001), interactive communication (path difference = 0.478, *p* = 0.001), and job pursuit intention (path difference = 0.501, *p* = 0.001). These results indicate that the negative associations between perceived AI-based interview experience and applicant outcomes were stronger among respondents in the Pakistani sample than among respondents in the Chinese sample.

However, the moderating effect of trust in AI did not differ significantly between the two samples. None of the path differences for the interaction terms were statistically significant (*p* > 0.05), as shown in Table 9. This indicates that trust in AI played a comparable moderating role in both samples. Overall, the MGA results support H7 by showing that cross-cultural differences emerged in the direct relationships between AI-based interviews and applicant outcomes, whereas the moderating role of trust in AI appeared comparable across the two cultural contexts examined.

**Table 9:** Multi-group comparison.

Path	Path diff. (China-Pakistan)	*P*-value (China-Pakistan)
AI-based interview ➔ perceived transparency	0.464	0.001
AI-based interview ➔ interactive communication	0.478	0.001
AI-based interview ➔ job pursuit intention	0.501	0.001
Trust in AI x AI-based interview ➔ perceived transparency	−0.009	0.854
Trust in AI x AI-based interview ➔ interactive communication	0.032	0.493
Trust in AI x AI-based interview ➔ job pursuit intention	−0.014	0.754

## 5 Discussion and conclusion

Drawing on a robust methodological and theoretical foundation, this study offers a comprehensive examination of cross-cultural differences and similarities in applicants’ responses to AI-based and human-based interview methods in China and Pakistan. Unlike the predominantly Western-focused literature, our research extends the cross-cultural scope to Asian contexts, providing novel insights into applicant perceptions in two distinct cultural environments.

The findings reveal that the negative association between perceived AI-based interview experience and perceived transparency, interactive communication, and job pursuit intention is significantly more pronounced among respondents in the Pakistani sample than respondents in the Chinese sample. This suggests that respondents in the Pakistani sample may perceive AI-driven recruitment processes as less transparent, less conducive to meaningful communication, and less motivating in terms of pursuing job opportunities. This pattern may reflect both lower familiarity with advanced recruitment technologies and a greater skepticism toward algorithmic decision-making in Pakistan, where face-to-face and relational interactions are traditionally valued. The significance of examining responses from the Pakistani sample to AI recruitment becomes particularly evident when considering the existing literature’s limited scope, which has predominantly focused on technologically advanced societies (Choung et al., 2023; Li, 2018).

This Western-centric approach has created a substantial knowledge gap regarding how emerging economies with distinct cultural values respond to algorithmic hiring practices. Our study addresses this limitation by providing empirical evidence from a South Asian context where interpersonal relationships and trust-building mechanisms play crucial roles in professional settings (Lee and Rich, 2021). The validation of these cultural preferences in recruitment contexts represents a significant contribution to the cross-cultural literature. Furthermore, while existing cross-cultural research on AI recruitment has primarily concentrated on East–West comparisons (Park and Jung, 2025), our Pakistan-China comparison offers valuable insights into intra-Asian variations that have been largely overlooked in academic discourse.

Conversely, respondents in the Chinese sample, who tend to have greater exposure to digital technologies and a higher degree of trust in institutional systems, reported relatively less negative perceptions of AI-based interviews. The value of exploring Chinese respondents’ attitudes toward AI recruitment is emphasized by China’s unique position as a rapidly digitizing economy with widespread AI adoption across various sectors (Qin et al., 2020). This digital transformation creates a distinctive cultural context where algorithmic decision-making may be more readily accepted compared to other Asian markets, making Chinese responses particularly relevant for understanding technology acceptance patterns. In addition, research on collectivistic cultures has consistently demonstrated higher acceptance of institutional authority and systematic processes (Park and Jung, 2025), and our findings extend these theoretical insights to AI-based recruitment contexts. While previous studies have shown that Chinese workers generally exhibit greater openness to digital innovations in workplace settings (Li, 2018; Yu et al., 2025), the specific application of these attitudes to recruitment processes remained unexplored prior to this investigation, highlighting the novel contribution of our research.

The role of trust in AI systems also emerged as a critical factor. While trust in AI was found to condition applicants’ perceptions in both samples, our multi-group analysis demonstrated that the moderating role of trust in AI systems is comparable between the Chinese and Pakistani samples. This finding is broadly consistent with research suggesting that trust is central to AI acceptance and may shape how individuals respond to AI-enabled systems (Gillespie et al., 2023). However, the present study does not claim that trust is universal or culturally neutral. Trust may itself be shaped by cultural, institutional, and technological conditions. Rather, the findings indicate that, within the two samples examined, higher trust in AI weakened negative reactions to AI-based interviews in a comparable way. In other words, within the Chinese and Pakistani samples studied, higher trust in AI systems similarly moderated applicants’ reactions to AI-based interviews, suggesting that fostering trust in technological tools may enhance the acceptance and perceived fairness of AI-driven recruitment in the two contexts examined.

Notably, this study highlights that while cultural context significantly influences how AI-based interviews are perceived, the moderating role of trust in AI appeared comparable across the two samples examined. This suggests that building trust in AI systems may be important for improving applicant responses to AI-based recruitment in both contexts, while broader claims about universality require further evidence from additional cultural settings.

### 5.1 Theoretical implications

This study advances the theoretical discourse on organizational justice, applicant reactions, and cross-cultural psychology by addressing the nuanced role of culture in responses to AI-based recruitment. By applying Bangerter et al. (2012) and Fairness Heuristic Theory Lind (2001), the research highlights the interpretive processes through which job applicants assess recruitment technologies across distinct cultural settings.

First, our findings expand the selection literature by explicitly integrating cultural context into the analysis of applicant reactions to AI-driven selection methods, a domain largely dominated by Western-centric research. The results demonstrate that justice perceptions, including perceived transparency and the opportunity for interactive communication, are not interpreted uniformly across cultural groups. Specifically, the study challenges the notion of universality in fairness perceptions by showing that applicants from different cultural backgrounds, such as China and Pakistan, ascribe different meanings to fairness signals in AI-mediated recruitment processes. This insight enriches the theoretical understanding of how organizational signals are received and decoded in cross-cultural contexts.

Second, the results emphasize the role of trust in AI systems as a key psychological mechanism influencing applicant reactions across cultures. Drawing on Fairness Heuristic Theory, the study shows that while the negative impacts of AI-based interviews differ between the two samples, the moderating function of trust in AI operates consistently in both contexts. However, consistent with our earlier theoretical discussion, we frame trust as culturally embedded in its level yet comparable in its moderating function, thereby offering conditional rather than universal support for trust-based accounts of technology acceptance.

Third, the results highlight that cultural context remains a critical factor in shaping applicant attitudes toward AI in recruitment. While both groups experience the novelty and efficiency of AI-based methods, only the Chinese sample demonstrates relative acceptance, likely due to higher exposure and trust in technology in their society. The pronounced negative reactions among Pakistani applicants reinforce the need to consider local cultural norms, expectations, and readiness for technological adoption when deploying AI in HR practices.

Finally, this research encourages further theoretical development by emphasizing the importance of integrating cultural and technological perspectives in models of applicant reactions. Our findings show that it is not simply the presence of AI in recruitment that matters, but how this technology is perceived and interpreted through cultural and trust-related lenses. This calls for a more nuanced, context-sensitive approach to studying and applying AI in personnel selection, with future research needed to explore evolving applicant attitudes as technology and cultural dynamics continue to change.

This study broadens the conceptual landscape of recruitment research by establishing that both cultural context and trust in AI are central to understanding applicant responses to AI-driven hiring processes. These theoretical insights highlight the need for organizations and researchers to account for both cultural differences and trust-building strategies when implementing AI-based selection systems globally.

### 5.2 Managerial implications

The present study delivers actionable guidance for senior management and HR professionals seeking to optimize AI-based interview systems across culturally diverse settings, such as China and Pakistan. Our results highlight that Pakistani applicants experience more pronounced negative effects in terms of perceived transparency, interactive communication, and job pursuit intention. This finding emphasizes the need for organizations to adapt AI-based interview protocols to cultures where interpersonal trust and human interaction are deeply valued. To address these cultural sensitivities, organizations should prioritize the integration of enhanced transparency measures. This includes offering comprehensive pre-interview briefings to explain AI system functionality, explicitly communicating evaluation criteria, and providing clear, accessible explanations regarding how AI-driven decisions are made. Such initiatives can help reduce skepticism and increase perceptions of fairness among applicants who may otherwise feel alienated by automated processes.

In contexts resembling the Chinese sample, where AI acceptance appears relatively higher, organizations are encouraged to focus on elevating the interactivity and engagement of AI-based interviews. Even when baseline trust and openness toward technology are strong, continuous improvement of candidate experience is essential to remain competitive. Enhancements might include real-time feedback, adaptive question flows, and responsive AI avatars that simulate authentic dialogue. These features not only maintain but can further enhance the organization’s image as an innovator and fair employer.

A consistent finding across both samples is the crucial role of trust in AI systems. Across the two contexts examined, trust was shown to weaken negative applicant reactions and improve candidate perceptions and intentions. To capitalize on this, organizations should develop core but locally adaptable trust-building strategies that include transparent communication about AI capabilities and limits, evidence of system reliability via pilot programs or case studies, strong assurances of data privacy and security, and clear demonstration of human oversight within the selection process. Because baseline trust itself varies across settings, these foundational elements should be locally tailored to accommodate specific cultural expectations rather than applied identically everywhere.

Our findings also indicate a pressing need for culturally competent HR practices. HR professionals should receive targeted training to understand how cultural differences shape applicant responses to AI-driven selection methods. This training should encompass not only awareness of technological and fairness perceptions but also practical skills for customizing the presentation and communication of AI systems to different cultural audiences, while upholding consistency in standards and employer branding. To ensure ongoing effectiveness, organizations should establish robust candidate experience monitoring systems. Regular tracking of trust levels, transparency perceptions, communication quality, and job pursuit intentions, disaggregated by cultural group, will enable timely identification of disparities and continuous refinement of recruitment technologies.

These practical implications suggest that successful AI-based recruitment implementation requires moving beyond one-size-fits-all approaches toward culturally-informed strategies that recognize and address distinct cultural responses to automated selection processes. Organizations that invest in such culturally-adaptive approaches are likely to achieve better candidate experiences, stronger talent pipelines, and more effective global recruitment outcomes.

### 5.3 Limitations and directions for future research

This study offers important insights into cross-cultural responses to AI-based recruitment, but is subject to several limitations. First, the findings are limited to Chinese and Pakistani contexts and may not generalize to other cultures. The authors did not directly measure cultural values or control for differences in technological infrastructure, digital literacy, or societal attitudes toward AI, which may have influenced the results. Future research should directly assess cultural dimensions and include more diverse settings to improve generalizability.

Second, and most importantly for interpreting the cross-cultural claims, participants in both countries were recruited through convenience, non-probability sampling. Therefore, the differences reported in this study cannot be treated as nationally generalizable estimates. The findings should be read as evidence about the sampled respondents rather than about the two national populations as a whole. Future studies should employ probability-based, stratified, or quota-based sampling strategies and include larger, more representative samples to permit stronger population-level inference.

Third, although trust in AI showed a comparable moderating role in both samples, evidence from only two countries cannot establish that this mechanism is universal. Accordingly, this study describes trust in AI as consistent across the two cultural contexts examined, rather than as a universal mechanism operating across all cultures. Future research should replicate the model across a wider range of countries, cultural-value profiles, and levels of technological development before making broader claims about universality. Studies that include many countries simultaneously or directly measure cultural-value dimensions at the individual level would be especially valuable for identifying the boundary conditions of trust in AI.

Fourth, the conceptualization of AI-based interviews in this study was relatively broad, which may limit the precision of the findings. AI interview technologies vary widely, ranging from asynchronous video interviews to real-time conversational agents and AI-supported decision systems. These different formats may produce different applicant responses. Future research should distinguish between specific types of AI-based interviews and examine whether applicant perceptions differ across these formats.

Fifth, this study did not investigate potential mediating variables, such as technology anxiety, digital self-efficacy, or emotional responses, which could further explain the relationship between interview type and applicant perceptions. Exploring these mediators could yield deeper insights, especially in cross-cultural contexts where individual differences are likely to be pronounced. Sixth, the study relied on cross-sectional survey data for hypothesis testing. Future studies should consider longitudinal designs to capture how applicants’ perceptions of AI-based recruitment, including transparency, interactive communication, trust, and job pursuit intention, may evolve over time or in response to organizational changes. Finally, a key limitation of this study is the relatively small sample sizes, with only 213 and 225 participants collected from China and Pakistan, respectively. Therefore, the generalizability of the findings should be interpreted with caution. Future research should aim to include larger and more diverse samples to enhance the robustness and applicability of the results across broader populations.

## Data Availability

The raw data supporting the conclusions of this article will be made available by the authors, without undue reservation.
